# Chronic Intermittent Hypobaric Hypoxia Restores Hippocampus Function and Rescues Cognitive Impairments in Chronic Epileptic Rats *via* Wnt/β-catenin Signaling

**DOI:** 10.3389/fnmol.2020.617143

**Published:** 2021-01-20

**Authors:** Can Sun, Jian Fu, Zhenzhen Qu, Lijing Jia, Dongxiao Li, Junli Zhen, Weiping Wang

**Affiliations:** ^1^Key Laboratory of Neurology of Hebei Province, Department of Neurology, The Second Hospital of Hebei Medical University, Shijiazhuang, China; ^2^Department of Neurology, Peking University Third Hospital, Beijing, China; ^3^Department of Emergency Surgery, The Second Hospital of Hebei Medical University, Shijiazhuang, China

**Keywords:** hypoxia, epilepsy, cognition, neurogenesis, hippocampus

## Abstract

Epilepsy is a complex neurological disorder with frequent psychiatric, cognitive, and social comorbidities in addition to recurrent seizures. Cognitive impairment, one of the most common comorbidities, has severe adverse effects on quality of life. Chronic intermittent hypobaric hypoxia (CIHH) has demonstrated neuroprotective efficacy in several neurological disease models. In the present study, we examined the effects of CIHH on cognition and hippocampal function in chronic epileptic rats. CIHH treatment rescued deficits in spatial and object memory, hippocampal neurogenesis, and synaptic plasticity in pilocarpine-treated epileptic rats. The Wnt/β-catenin pathway has been implicated in neural stem cell proliferation and synapse development, and Wnt/β-catenin pathway inhibition effectively blocked the neurogenic effects of CIHH. Our findings indicate that CIHH rescues cognitive deficits in epileptic rats *via* Wnt/β-catenin pathway activation. This study establishes CIHH and Wnt/β-catenin pathway regulators as potential treatments for epilepsy- induced cognitive impairments.

## Introduction

Epilepsy is a diverse group of neurological disorders characterized by recurrent hyperactivation of neural circuits, manifesting as seizures (Pearson et al., 2015). Temporal lobe epilepsy (TLE) is one of the most frequent and severe types of epilepsy (Leyden et al., 2015). It is characterized by hippocampal degeneration and scar formation (sclerosis), spontaneous recurrent seizures (SRS), and antiepileptic drug (AED) resistance (Peixoto-Santos et al., 2015). Recurrent seizures often result in neuronal loss, deficient synaptic plasticity, and ensuing cognitive impairments that markedly degrade patient quality of life (Tavakoli et al., 2011; Helmstaedter and Witt, 2017). Moreover, AEDs that suppress seizures can have adverse effects on cognitive function (Witt et al., 2015). Therefore, it is important to identify novel interventions that not only treat epilepsy but also mitigate cognitive impairments.

Severe hypoxia can lead to neurodegeneration, while mild hypoxia may have neuroprotective effects that result in brain tolerance to multiple stressors (termed pre-conditioning) (Rybnikova and Samoilov, 2015). Mild hypoxia has been shown to promote neural stem cell (NSC) self-renewal, regulate the differentiation state of NSCs *in vitro* (De Filippis and Delia, 2011), and induce neurogenesis in the dentate gyrus (DG) of adult rats *in vivo* (Zhu et al., 2010; Zhang et al., 2015). Neurogenesis is crucial for hippocampus functions, including spatial learning and object recognition (Deng et al., 2010; Koehl and Abrous, 2011; Marin-Burgin and Schinder, 2012). Oxygen and NSC homeostasis are also involved in the maintenance of synaptic plasticity and circuitry in the brain8. Hypoxia enhances the transcription of genes associated with presynaptic development (Curristin et al., 2002).

Our previous study suggested that chronic intermittent hypobaric hypoxia (CIHH) suppresses seizures and rescues epilepsy-induced spatial learning and memory impairments (Zhen et al., 2014; Sun et al., 2019), but the underlying mechanisms remain elusive. The present study further evaluated the effects of CIHH on epileptic rats and investigated the underlying mechanisms.

## Materials and Methods

### Animals and Groups

Experiments were performed in accordance with the guidelines for the Care and Use of Laboratory Animals published by the US National Institutes of Health and were approved by the Second Hospital of Hebei Medical University (Animal licenses no.: SCXK [Hebei 2018-004]). Male Sprague Dawley (SD) rats (6–8 weeks old, 180–200 g) were purchased from Beijing Vital River Laboratory Animal Technology Company and housed under a 12/12 h light/dark cycle, 25 ± 1°C ambient temperature, and 50–60% humidity. Animals were provided *ad libitum* access to food and water and acclimatized to the laboratory environment for 1 week prior to experiments. Rats were divided into two populations, one to study the effects of CIHH on hippocampal neurogenesis and synaptic plasticity, and the other to study the molecular mechanisms of CIHH using the Wnt/β-catenin antagonist Dickkopf-1 (DKK-1). The first population was randomly divided into three groups: Control, Pilocarpine-treated (Pilo), and Pilo + CIHH. The second was randomly divided into four groups: Control, Pilo, Pilo + CIHH, and Pilo + CIHH + DKK-1.

### Pilocarpine Treatment

Lithium pilocarpine was used to induce status epilepticus (SE) as described 16. Briefly, rats were administrated lithium chloride (127 mg/kg, i.p.) and then a single dose of pilocarpine (50 mg/kg, i.p.; Sigma) 18 h later to induce SE. If SE did not occur within 30 min, the same pilocarpine dose was re-administered and repeated up to five times in total. Atropine (1 mg/kg) was injected 30 min before the first pilocarpine administration to prevent peripheral effects.

### CIHH Protocol and BrdU Incorporation *in vivo*

**Figure 1:**
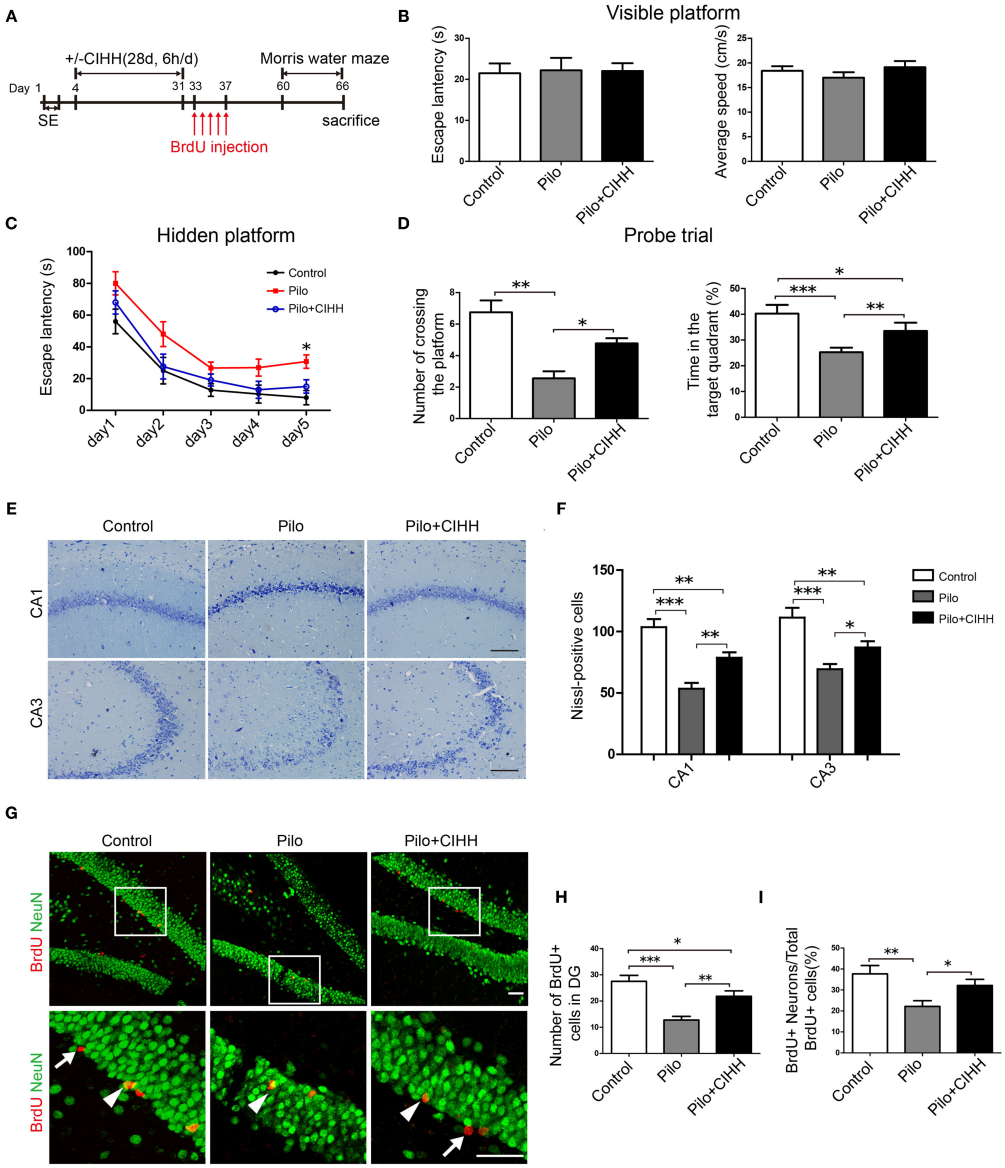
CIHH improves spatial learning and promotes hippocampal neurogenesis in pilocarpine-treated epileptic rats. **(A)** The experimental paradigm for examining the effects of CIHH treatment on cognitive deficits in chronic epileptic rats. The spatial learning capacity of each group was tested using the Morris water maze (MWM) **(B–D)**. **(B)** A visible platform test was conducted to control for group differences in vision and swim speed. **(C)** Escape latency over 5 days was recorded in the hidden platform tests. *P < 0.05 by repeated measures ANOVA. **(D)** A probe trial without the hidden platform was conducted after 5 days of training. *P < 0.05, **P < 0.01, ***P < 0.001 by ANOVA. Control (*n* = 9 rats), Pilo (*n* = 9), Pilo + CIHH (*n* = 9). **(E,F)** Nissl-stained brain sections in CA1 and CA3 subregions representing live cells in each groups. *P < 0.05, **P < 0.01, ***P < 0.001 by one-way ANOVA. **(G)** Double immunofluorescence images of NeuN (green, neuronal marker) and BrdU (red, proliferative cell marker) in the DG region of the hippocampus. The number of BrdU+ cells (white arrows) in the DG **(H)** and the proportion of BrdU+/NeuN+ cells (white arrowheads) to the total number of BrdU+ cells **(I)** were reduced by epilepsy and rescued by CIHH. *P < 0.05, **P < 0.01, ***P < 0.001 by one-way ANOVA. All values are presented as the mean ± SEM (*n* = 3 rats/group). **(E)** Scale bar, 100 μm. **(G)** Scale bar, 50 μm.

Starting 3 days after SE was induced, rats were placed in a chamber for intermittent hypobaric hypoxic exposure as described previously (Sun et al., 2019). While in the chamber, air pressure was gradually decreased to that at an altitude of 3,000 m (PB = 525 mmHg; PO_2_ = 108.8 mmHg). Animals were exposed to CIHH 6 h per day for 28 consecutive days. Animals in the Control and Pilo groups were placed in the chamber in which room air was circulated for the corresponding periods. Starting the second day after CIHH treatment, BrdU (50 mg/kg, i.p.; Sigma) was injected into rats for 5 consecutive days to label proliferating cells. Rats were sacrificed 28 days after the last BrdU injection. Behavioral tests were conducted prior to sacrifice. The timeline of experimental procedures is shown in Figure 1A.

### Isolation of Rat Hippocampus NSC and Hypoxia Treatment *in vitro*

Primary NSCs were prepared from fetal SD rats (E18) as previously described 16. Subsequently, third-generation NSCs were divided into three groups, normoxia, hypoxia, and hypoxia + DKK-1. Cells in the normoxia group were placed in a conventional incubator with 75% N_2_, 5% CO_2_, and 20% O_2_. For hypoxic culture, cells were maintained in a gas mixture composed of 90% N_2_, 5% CO_2_, and 5% O_2_. To block Wnt signaling, the Wnt/β-catenin inhibitor DKK-1 (100 ng/ml) was added with every medium change during hypoxic growth.

To analyze the effects of hypoxia on NSC proliferation, BrdU (10 μmol/L) was added into the medium 5 days after hypoxia exposure for 36 h. The normoxic group received the same treatments except in a normoxic environment. Cells were fixed in 4% paraformaldehyde (PFA) and incubated with 2 N HCl at 37°C for 30 min. After washing in PBS, cells were examined by immunofluorescence staining as described below.

### Neuronal Differentiation Under Hypoxia

To induce neuronal differentiation, third-generation NSCs were plated onto poly-D-lysine-coated coverslips in neurobasal medium containing 2% B27 supplement (Gibco) and 1% penicillin/streptomycin. Differentiated NSCs were divided into three groups, normoxia, hypoxia, and hypoxia + DKK-1. The hypoxia protocol and DKK-1 treatment were the same as described above. After 10 days of differentiation under hypoxia or normoxia, cells were fixed in 4% PFA and subjected to immunofluorescence staining.

### Stereotaxic Surgery and DKK-1 Injection

Before CIHH treatment, rats were anesthetized with sodium pentobarbital (0.84 mL) and placed in a stereotaxic instrument. A small hole was drilled into the skull and a stainless-steel guide cannula (RWD) was inserted into the DG (AP −4.0 mm, Lat 2.5 mm, DV 3.6 mm) and anchored to the skull with dental acrylic and screws. Animals were injected with DKK-1 (1 μg/3 μl) or equal-volume sterile saline (vehicle) at 0.6 μl/min once per week during the 28-days CIHH treatment period. Behavioral assessments were performed the day after the last DKK-1 administration.

### Behavioral Assessments

Spatial learning and memory were assessed by the Morris water maze (MWM) test as our previously described (Sun et al., 2019). Next, a Novel object recognition (NOR) test was performed to assess non-spatial short-term memory. Rats were habituated to a testing chamber (50 × 50 × 50 cm) with one object for 5 min. The next day, rats were placed in a testing chamber to explore two identical objects (object A1 and A2) for 5 min. After 24 h, one familiar object A was replaced with a novel object (object B). Rats were recorded exploring objects A and B for 5 min. The total times contacting objects A and B were recorded as TA and TB, respectively. A recognition index was calculated using the following formula: [TB]/[TA + TB].

### Tissue Preparation

Rats were anesthetized and transcardially perfused with cold PBS prior to harvesting brain tissues for western blot analysis. For immunostaining and Nissl staining, rats were perfused with cold PBS followed by 4% PFA. Fixed brains were then post-fixed in 4% PFA for 24–48 h at 4°C. Brains from rats receiving BrdU were cut into 30 μm free-floating sections using a vibratome. Other fixed tissues were dehydrated, embedded in paraffin, cut into 5 μm sections, and prepared for Nissl staining and immunohistochemistry.

### Nissl Staining, Immunochemistry, and Immunofluorescence Staining

Sections were dewaxed in xylene and rehydrated in graded ethanol. Subsequently, sections were stained with 1% thionine solution for 5 min, rinsed and dehydrated in graded ethanol. The sections were cleared in xylene and mounted with neutral balsam. Brain sections were examined using a light microscope. Neuronal survival in the CA1 and CA3 subfields of the hippocampus was quantified using Image Pro Plus (IPP) 6.0 software.

For immunochemistry, the paraffin sections were deparaffinized, rehydrated, and subjected to antigen retrieval (95°C, 10 min). Sections were rinsed in PBS, incubated in 3% H_2_O_2_, blocked in 10% goat serum for 30 min at 37°C, and incubated overnight at 4°C with primary antibody against doublecortin [DCX, Cell Signaling Technology (CST)]. After washing, sections were incubated with a secondary and third antibody for 15 min each. Immunostaining was visualized using a DAB detection kit (ZLI-9018, China). The number of DCX-positive cells in the granule cell layer of the DG was quantified using IPP 6.0 software.

For immunofluorescence staining, cells or tissue sections were fixed with 4% PFA, incubated in 2 N HCl at 37°C for 30 min, and neutralized in boric acid. After blocking and permeabilizing with 10% goat serum plus 0.3% Triton X-100, the sections were treated with one of the following primary antibodies as indicated: anti-BrdU (555627, BD Biosciences, USA), anti-NeuN (ABN78, Millipore, USA), anti-Nestin (ab92391, Abcam), anti-DCX (4606, CST), or anti-Wnt3a (09-162, Millipore). After overnight incubation at 4°C, sections were incubated in secondary antibodies conjugated to Alexa 488 or 594 (KPL, USA) for 1 h at 37°C. After washing in PBS and counterstaining with DAPI, sections were mounted on slides for imaging under a confocal laser-scanning microscope (Olympus FV1000S, Japan).

BrdU-positive cells in the DG subgranular zone (SGZ) were counted blindly in eight 30-μm coronal sections spaced 180 μm apart for each animal. The neuronal phenotype of BrdU-positive cells was confirmed by colocalization with NeuN signals using Z-plane sectioning. Data were obtained from eight sections per rat and six rats per treatment group.

### Golgi-Cox Staining

According to the manufacturer's instructions with the Golgi-Cox OptimStain Kit (HTKNS1125, USA), brains were removed and sliced into blocks of 10 mm thickness containing the hippocampus. Sections were rinsed and immersed in impregnation solution. The solution was replaced the next day and tissues stored for 2 weeks in the dark. Tissues were then transferred into Solution-3 for 24 h. Brain sections (100 μm) were prepared using a vibratome and mounted on gelatin–coated slides with Solution-3. After drying naturally, slices were stained with a mixture of Solution-4, Solution-5, and ddH_2_O for 10 min, followed by gradient dehydration ethanol. Thereafter, the samples were cleared in xylene and mounted. Secondary and tertiary apical dendrites within the subregions of hippocampus were imaged and analyzed using ImageJ (USA). Dendritic spine density was calculated as the number of spines per μm of dendrite. Total dendritic length was calculated as the sum of the primary, secondary, and tertiary dendritic lengths. Dendritic complexity was assessed by the Sholl method.

### Electron Microscopy

Rats were anesthetized and transcardially perfused with cold PBS followed by 4% PFA plus 4% glutaraldehyde (GA). Brains were removed and tissue blocks containing the hippocampal CA1 area (1 × 1 × 1 mm^3^) were separated, post-fixed in 4% GA, and stained with 1% osmium tetroxide. Following dehydration in graded ethanol and propylene oxide, tissues were embedded in resin. Ultrathin sections were cut on an ultramicrotome and stained with uranyl acetate and lead citrate. Synaptic structures were observed. Post-synaptic density (PSD) thickness and synaptic cleft width were measured using ImageJ. The technique proposed by Guldner and Ingham (1980) was adopted here for the estimation of PSD thickness, while synaptic cleft width was measured using a multi-point averaging method (Luo et al., 1991).

### Long Term Potentiation (LTP) Recording

Rats were anesthetized and placed in a stereotaxic apparatus for field excitatory post-synaptic potential (fEPSP) recording. A combined simulating/recording electrode was inserted into the hippocampal CA1 region, with the tip of the recording electrode 4.0-mm posterior to bregma and 3.8-mm lateral to the midline. Baseline fEPSPs of 30–50% maximum were elicited at 0.033 Hz for at least 20 min, then high-frequency stimulation (HFS, three trains of 20 stimuli delivered at 200 Hz with an intertrain interval of 30 s) was applied to induce LTP. After HFS, fEPSPs were recorded at 0.033 Hz for 1 h. The amplitudes of post-HFS fEPSPs were normalized to the average baseline fEPSP amplitude and averaged across animals. After electrophysiological recordings, hippocampi were isolated and stored at −80°C for western blotting.

### Western Blotting

Western blotting was performed as previously reported 16. Separated proteins were immunolabeled with antibodies against synaptotagmin (Syt) (ab13259; Abcam), PSD-95 (3450s; Cell Signaling Technology), Kalirin-7 (07-122; Millipore), Wnt3a (09-162, Millipore), β-catenin (51067-2-AP, Proteintech), p-GSK-3β (9323, CST), GSK-3β (12456, CST), and CyclinD1 (ab137875, Abcam). Blots were imaged using an Odyssey IR fluorescence scanning system, and protein expression quantified by densitometric analysis using ImageJ. Anti-β-actin (Proteintech, China) was used as an internal gel-loading control.

### Statistical Analysis

All values are shown as the mean ± standard error of the mean (SEM). Escape latencies in the MWM and number of dendritic intersections were compared among groups by repeated measures ANOVA. Other datasets were compared by one-way ANOVA followed by a *post-hoc* test, either the LSD test or Dunnett's T3 test. All statistical analyses were performed using SPSS 22.0 (USA) or GraphPad Prim 5.0 software (USA). A *P* < 0.05 (two-tailed) was considered statistically significant.

## Results

### CIHH Alleviates Cognitive Impairments and Promotes Hippocampal Neurogenesis in Epileptic Rats

To evaluate the effect of CIHH treatment on spatial learning and memory ability in epileptic rats, we compared MWM performance among Control, Pilo, and Pilo + CIHH groups. A visible platform test (Figure 1B) revealed no differences in vision or swimming speed among groups. In the hidden platform test, Pilo group (epileptic) rats exhibited longer escape latencies on training day 5 compared to Control and Pilo + CIHH rats, suggesting that CIHH can prevent spatial learning deficits caused by epilepsy (Figure 1C). In the probe trial, Pilo + CIHH rats demonstrated a greater number of crossings over the previous platform location and spent a greater proportion of swim time in the target quadrant than Pilo group rats (Figure 1D), suggesting that CIHH can also mitigate the deleterious effects of epilepsy on spatial memory.

Next, we examined neuronal death in the hippocampus. Nissl staining revealed significant neuronal loss and damaged neuronal structure in CA1 and CA3 subregions of epileptic rat, such as irregularly aligned, shrunken somata, and pyknotic nuclei (Figures 1E,F). Pilo + CIHH group rats exhibited less severe neuronal loss and better more integrated cell morphology. Therefore, epilepsy- induced hippocampal neuron injury was prevented or injured neurons replaced in rats subjected to CIHH. BrdU incorporation and NeuN staining indicated fewer adult-born cells in the DG granule cell layer of epileptic rats compared to control rats (Figures 1G,H), while CIHH treatment reversed this decrease. CIHH also increased the proportion of BrdU+ neurons to total BrdU+ cells (Figure 1I). Thus, CIHH induced adult hippocampal neurogenesis and promoted newborn cells to differentiate into neurons.

### CIHH Enhances Structural Synaptic Plasticity and Upregulates Wnt/β-Catenin Pathway in the Hippocampus of Epileptic Rats

**Figure 2:**
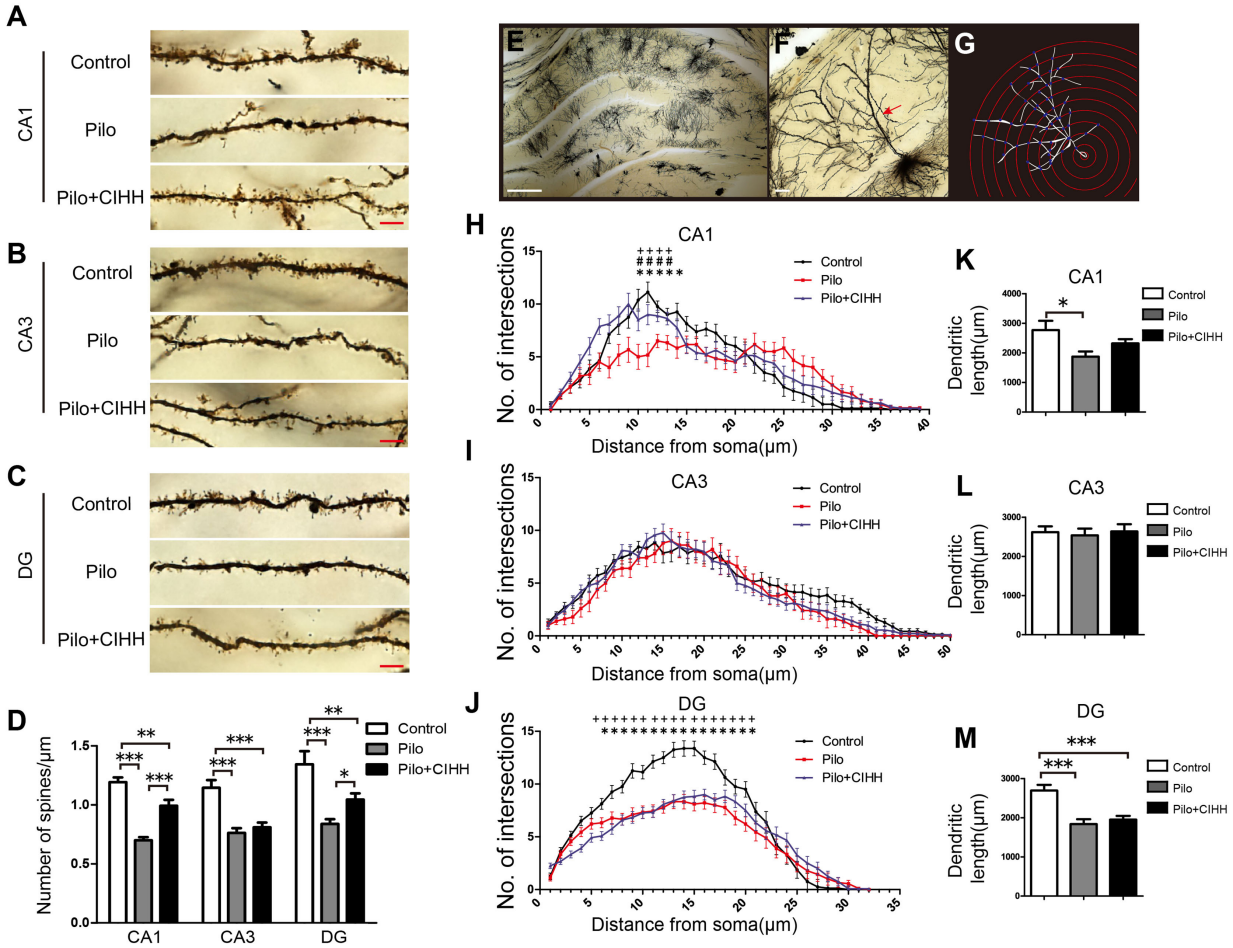
CIHH increases dendritic spine density and dendritic complexity in the hippocampus of epileptic rats. Golgi staining was used to reveal dendritic spines from secondary and tertiary apical dendrites in the CA1 **(A)**, CA3 **(B)**, and DG **(C)** regions of the hippocampus as summarized in **(D)**. **(E)** Image of the hippocampus showing a single Golgi-impregnated neuron. **(F)** Apical dendrites (red arrow). **(G)** Concentric circles used for Sholl analysis. **(H–J)** Mean numbers of apical dendritic intersections in the CA1 **(H)**, CA3 **(I)**, and DG **(J)** regions of the hippocampus as assessed by Sholl analysis. Pilo vs. Control, *P < 0.05; Pilo + CIHH vs. Control, ^+^*P* < 0.05; Pilo + CIHH vs. Pilo, ^#^*P* < 0.05 by repeated-measures ANOVA. **(K–M)** Graphs showing total apical dendritic length (μm) in the CA1, CA3, and DG regions of the hippocampus. *P < 0.05, **P < 0.01, ***P < 0.001 by one-way ANOVA. All values are presented as the mean ± SEM. *n* = 3 rats/group. **(A–C)** Scale bar, 5 μm. **(E)** Scale bar, 200 μm. **(F)** Scale bar, 20 μm.

Structural and functional synaptic plasticity are widely considered neurocellular mechanisms for learning and memory (Richter and Klann, 2009). To explore the contribution of structure plasticity to improved spatial memory following CIHH, we examined dendritic morphology and synaptic ultrastructure. Golgi staining revealed a lower density of dendritic spines in hippocampal CA1, CA3, and DG subregions of epileptic rats compared to control rats (Figures 2A–D), while Pilo + CIHH group rats demonstrated a higher density of dendritic spines in CA1 and DG than epileptic rats. Alternatively, CIHH had no effect on dendritic spine density in CA3. Sholl analysis of dendritic intersections as a function of distance from the soma (Figures 2E–G), revealed a significant decrease in dendritic complexity in CA1 and DG areas of epileptic rats compared to control rats (Figures 2H,J), while CIHH reversed this decrease in the CA1 but not the DG. Total apical dendrite length was also reduced in CA1 and DG of epileptic rats compared to control rats (Figures 2K,M), but this effect was not reversed by CIHH treatment. There were no significant differences in the number of dendritic intersections or total dendritic length among groups in CA3 (Figures 2I,L). Nonetheless, these findings indicate that CIHH can partially mitigate deficient dendritic structural plasticity induced by epilepsy in hippocampal CA1 and DG.

**Figure 3:**
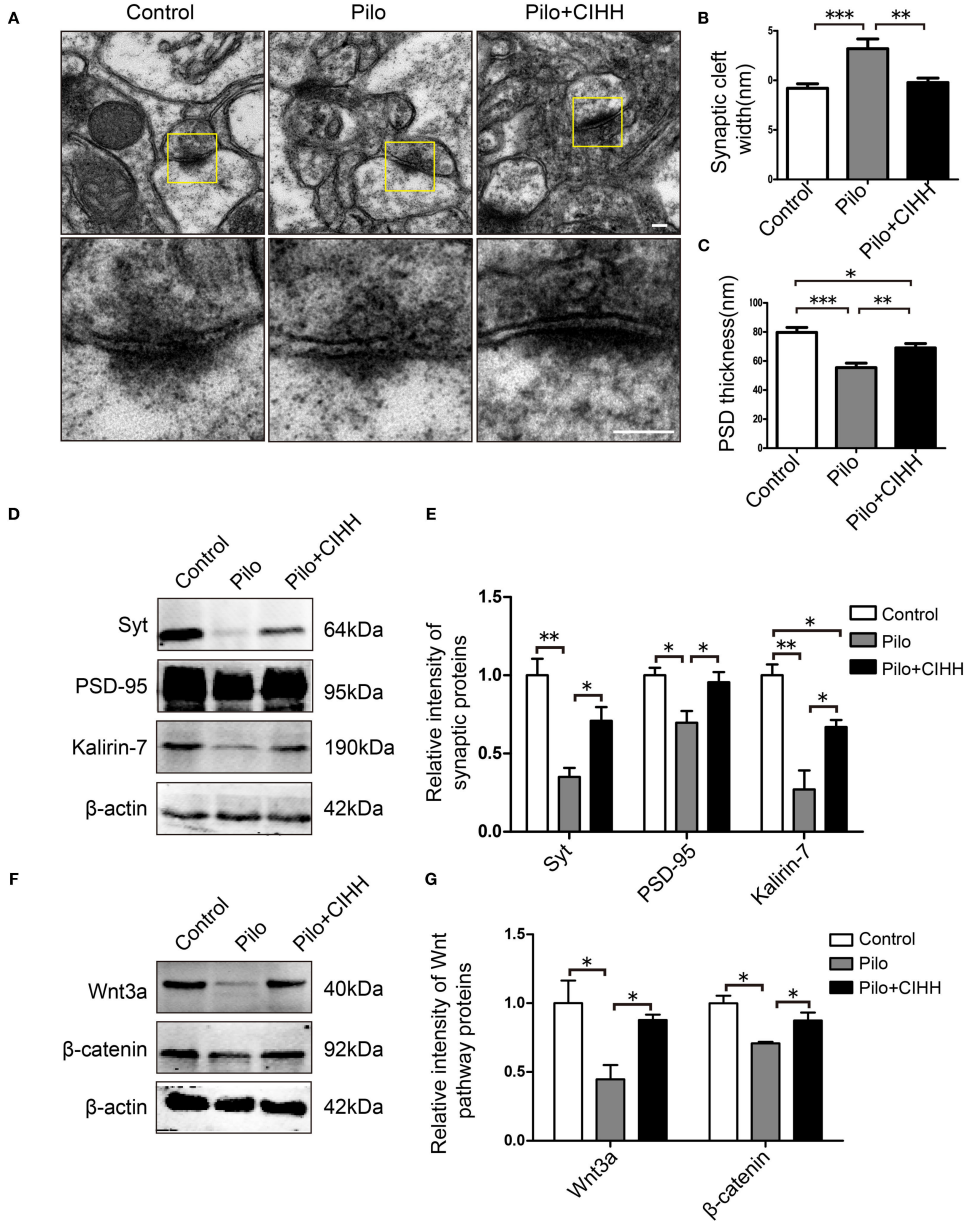
Effects of CIHH on synaptic ultrastructure in the CA1 and plasticity-related protein expression in the hippocampus. **(A)** Scanning electron microscope images showing synaptic ultrastructure. Synaptic cleft width **(B)** and PSD thickness **(C)** in each group. **(D,E)** Western blots of Syt, PSD-95, and kalirin-7 expression in the hippocampus. **(F,G)** Western blots of Wnt3a and β-catenin expression in the hippocampus. All values are normalized to the control samples. All values are presented as the mean ± SEM (*P < 0.05, **P < 0.01, ***P < 0.001 by one-way ANOVA). *n* = 3 rats per group. **(A)** Scale bar, 100 nm.

Epileptic rats also exhibited a significant increase in synaptic cleft width and a decrease in PSD thickness among CA1 synapses compared to control rats, and these changes were rescued by CIHH (Figures 3A–C). Additionally, CIHH reversed the epilepsy-induced downregulation of synaptic plasticity-associated proteins Syt, PSD-95, and Kalirin-7 (Figures 3D,E). These results suggest that CIHH improves hippocampal synaptic plasticity after epilepsy.

We then examined expression levels of Wnt/β-catenin pathway proteins in the hippocampus by western blotting. Expression levels of Wnt3a and β-catenin were significantly reduced in epileptic rats compared to control rats, and downregulation of both proteins was reversed by CIHH (Figures 3F,G). Therefore, we speculated that CIHH improves cognitive function in epileptic rats by activation of Wnt/β-catenin pathway.

### Wnt/β-Catenin Pathway Blockade Results in Cognitive Impairments in CIHH-Treated Epileptic Rats

**Figure 4:**
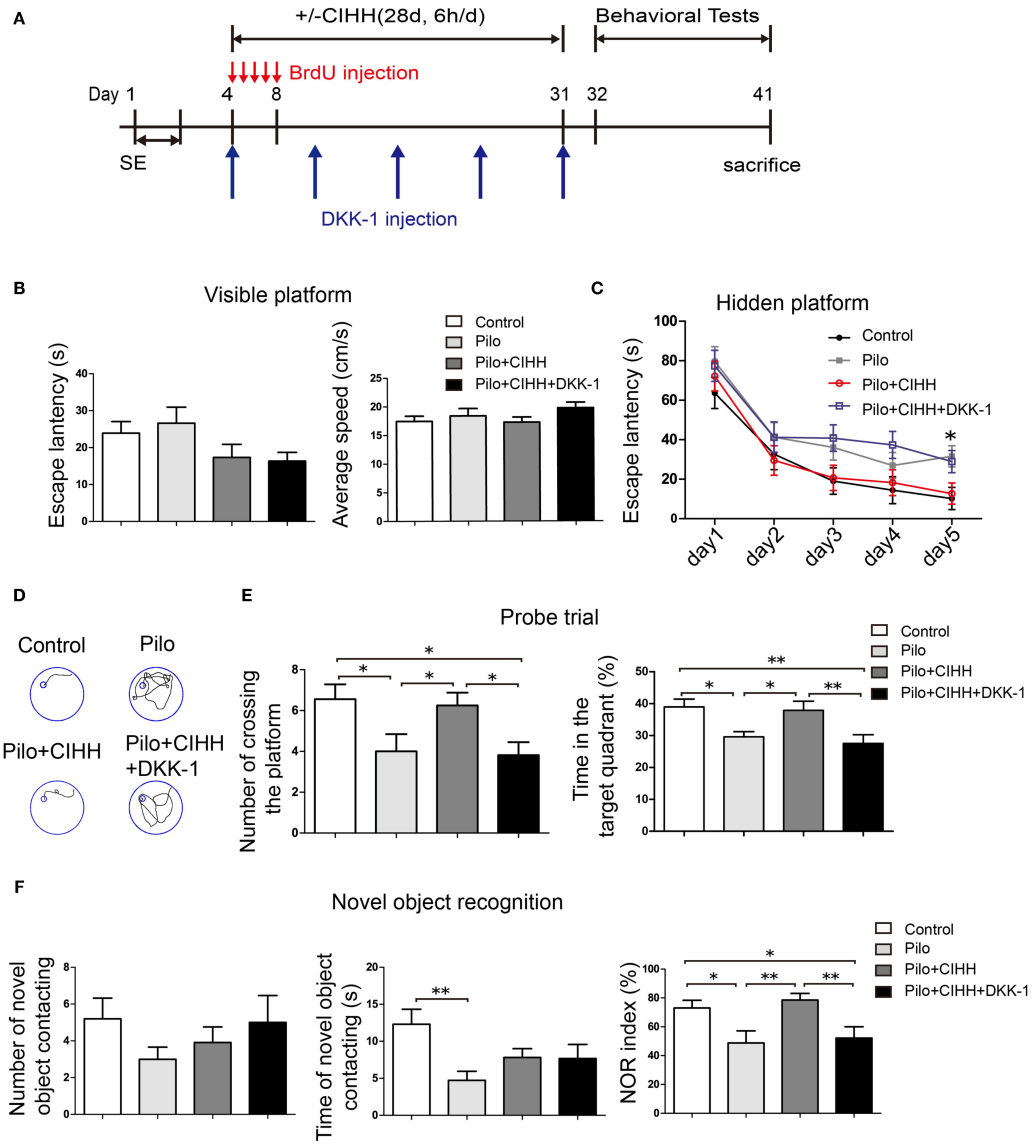
Improved spatial memory and novel object recognition (NOR) in epileptic rats by CIHH is abolished by Wnt signaling blockade. **(A)** Visible platform trials. There were no differences in escape latency and average swimming speed among groups. **(B)** Hidden platform tests showing significantly prolonged escape latencies in the Pilo + CIHH + DKK-1 group on day 5 compared to the Pilo + CIHH group. *P < 0.05 by repeated measures ANOVA. **(C)** Typical swimming tracks on the last day of hidden platform trials. **(D,E)** Results of the probe trial conducted after 5 days of training. **(F)** NOR test was conducted in four groups. All values are presented as the mean ± SEM. *P < 0.05, **P < 0.01 by one-way ANOVA. Control (*n* = 11 rats), Pilo (*n* = 12), Pilo + CIHH (*n* = 12), Pilo + CIHH + DKK-1 (*n* = 11).

To investigate the contribution of Wnt/β-catenin signaling to cognitive improvement following CIHH in epileptic rats, we injected epileptic rats with the Wnt/β-catenin antagonist DKK-1 or vehicle during CIHH treatment (i.e., Pilo + CIHH + DKK-1 and Pil + CIHH groups) and examined hippocampus-dependent spatial learning, spatial memory and non-spatial recognition memory. In the MWM test, there were no differences in escape latency and average speed in the visible platform tests among groups (Figure 4A), thereby excluding the impact of vision and swimming ability on group differences. On the 5th day of hidden platform trials, the Pilo + CIHH + DKK-1 group exhibited a significantly longer mean escape latency compared to the Pilo + CIHH (vehicle-injected) group and the Control group, but no differences compared to the Pilo group (Figures 4B,C). In the probe trial, both the number of platform crossings and proportion of time in the target quadrant were significantly lower in the Pilo + CIHH + DKK-1 group compared to the Pilo + CIHH group (Figure 4D). These results suggest that Wnt pathway contributes to the rescue of impaired spatial learning and memory by CIHH in epileptic rats.

In the NOR test, there were no group differences in the number of novel object contacts among groups, but the duration of novel object contact was lower in the Pilo group than the Control group, suggesting that epilepsy also impairs object recognition (Figure 4E). Contrary to expectations, however, these metrics did not differ among Pilo, Pilo + CIHH, and Pilo + CIHH + DKK-1 group animals. As group differences in NOR may be obscured by individual differences in activity, we compared the novel object recognition index, which controls for overall object contact propensity. Indeed, the object recognition index was reduced in the Pilo group compared to the Control group, and CIHH treatment reversed this decrease. Furthermore, DKK-1 reduced the object recognition index compared to the Pilo + CIHH group. These findings again indicate that Wnt pathway contributes to the rescue of NOR by CIHH in epileptic rats.

### Wnt/β-catenin Pathway Blockade by DKK-1 Inhibits Hypoxia- Induced Hippocampal Neurogenesis *in vivo* and *vitro*

**Figure 5:**
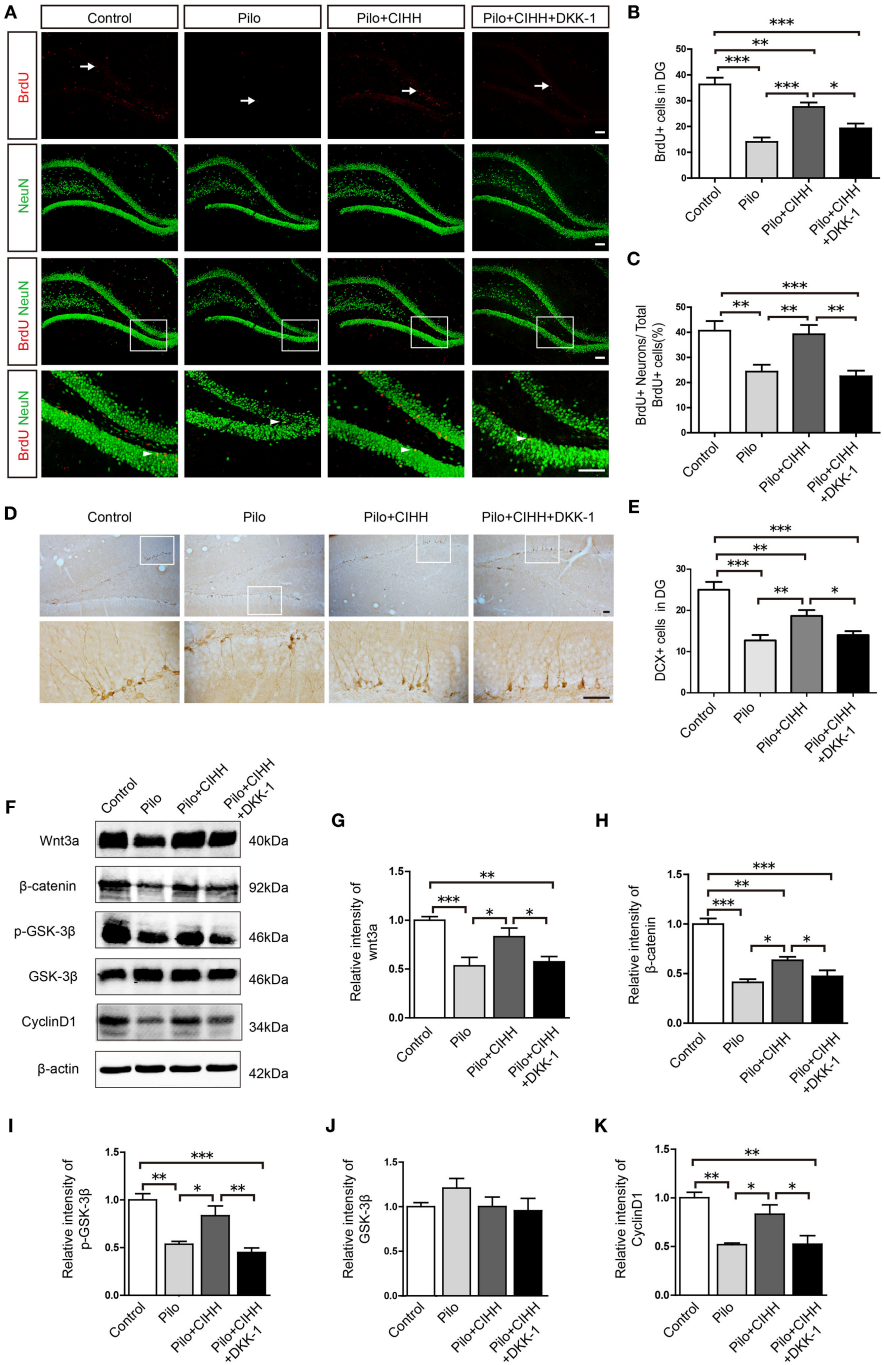
The Wnt inhibitor DKK-1 reduces CIHH-induced hippocampal neurogenesis and downregulates Wnt/β-catenin pathway protein expression in epileptic hippocampi. **(A)** Representative images of BrdU (red)/NeuN (green) immunostained cells in the DG of each group. White arrows indicate BrdU+ cells and white arrowheads indicate BrdU+/NeuN+ cells. **(B)** The number of BrdU+ cells in the DG was reduced by epilepsy and rescued by CIHH but not by CIHH + DKK-1. **(C)** Proportion of BrdU+/NeuN+ cells to the total number of BrdU+ cells (%) showing a similar pattern. **(D)** Representative DG cells immunostained with the immature neuronal marker DCX from each group. **(E)** The number of DCX+ cells in the DG region was reduced by epilepsy and rescued by CIHH but not by CIHH + DKK-1. **(F)** Representative images of western blots for Wnt/β-catenin pathway protein expression levels in the hippocampus. **(G–K)** Relative intensities of Wnt and β-catenin expression in the hippocampus. All values are normalized to the control samples and reveal suppression by epilepsy and rescue by CIHH but not by CIHH + DKK-1. All values are presented as the mean ± SEM. *P < 0.05, **P < 0.01, ***P < 0.001 by one-way ANOVA. *P < 0.05, **P < 0.01, ***P < 0.001. *n* = 3 rats per group. **(A)** Scale bar, 100 μm. **(D)** Scale bar, 50 μm.

Neurogenesis is critical for the maintenance of hippocampal function. We demonstrated that CIHH promotes hippocampal neurogenesis and Wnt signaling-dependent cognitive rescue in epileptic rats. We therefore examined the effect of DKK-1 injection into the DG on CIHH-mediated neurogenesis. Indeed, DKK-1 injection during CIHH induced a significant decline in the number of BrdU+ cells in the DG compared to vehicle-injected CIHH-treated rats (Figures 5A,B). Similarly, the proportion of BrdU+ neurons among the total number of BrdU+ cells was reduced by DKK-1 injection during CIHH (Figures 5A,C). Furthermore, the number of DCX+ cells in the DG, represent for immature neurons, was significantly reduced in the Pilo + CIHH + DKK-1 group compared to the Pilo + CIHH group (Figures 5D,E). These results indicate that Wnt signaling contributes to CIHH-induced hippocampal neurogenesis and neuronal differentiation in the presence and absence of epilepsy. In addition, CIHH upregulated and DKK-1 inhibited expression of hippocampal Wnt signaling proteins Wnt3a, β-catenin, and p-GSK-3β as well as expression of the downstream target protein CyclinD1 (Figures 5F–K), confirming that our local DKK-1 injection protocol effectively inhibited Wnt pathway in the hippocampus.

**Figure 6:**
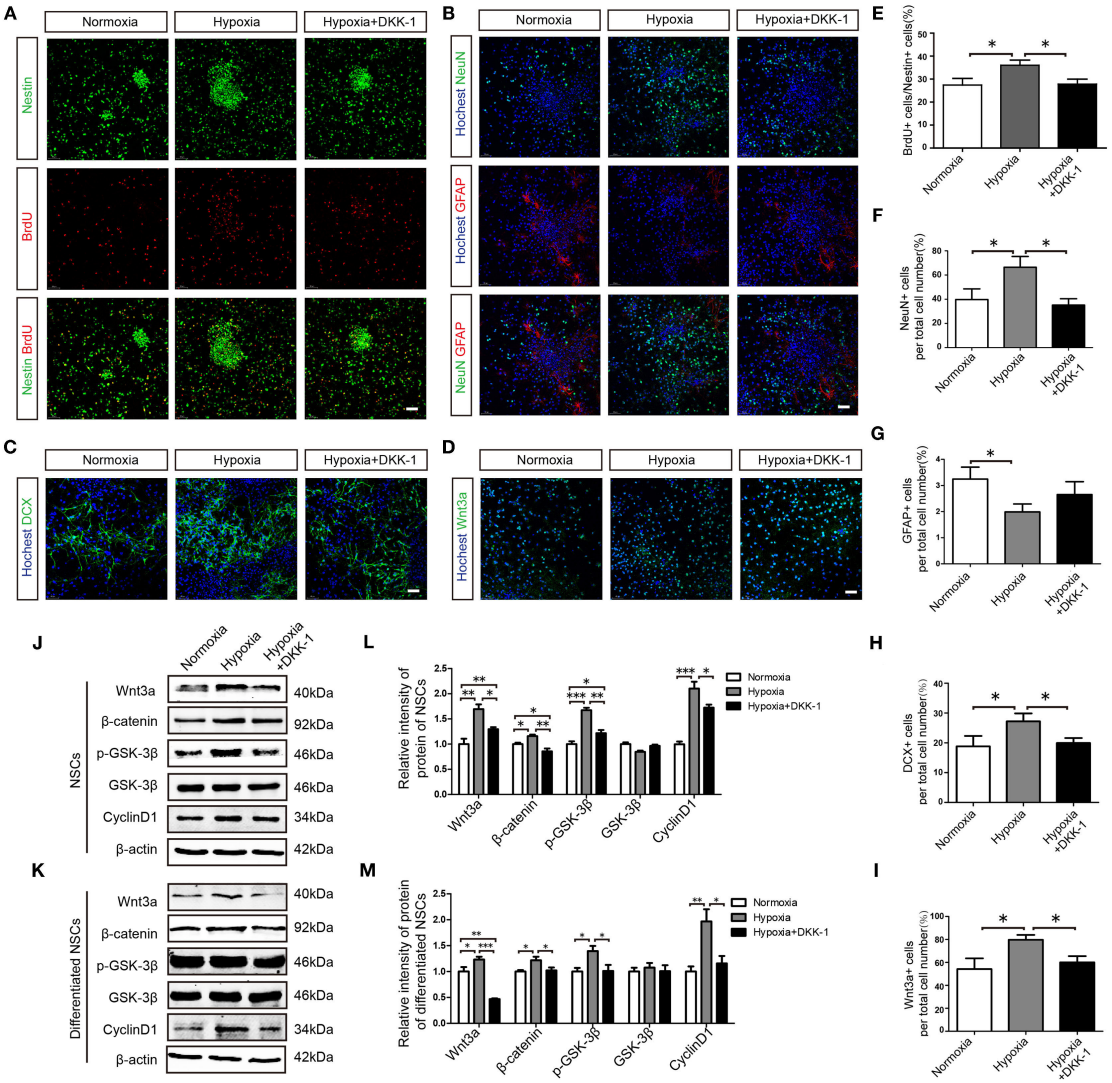
Hypoxia promotes hippocampus-derived NSC proliferation and neuronal differentiation *via* the Wnt/β-catenin pathway. **(A)** Effects of hypoxia on NSC proliferation were examined by Nestin and BrdU staining. **(B)** Differentiation of NSCs was assessed by NeuN and GFAP. **(C)** Neuronal differentiation of NSCs was assessed by the expression of the immature neuron marker DCX. **(D)** The effects of hypoxia on Wnt3a expression in differentiated NSCs. The proportion of BrdU+ cells to Nestin+ cells (%) in proliferated NSCs **(E)** and the ratio of NeuN+ cells among total cells (%) in differentiated NSCs **(F)** were enhanced by hypoxia but not by hypoxia+ DKK-1. The ratio of GFAP+ cells among total cells (%) **(G)** was reduced by hypoxia. The ratio of DCX+ cells among total cells (%) **(H)** and the ratio of Wnt3a+ cells among total cells (%) **(I)** were enhanced by hypoxia but not by hypoxia + DKK-1. Western blots of Wnt/β-catenin pathway proteins expression in proliferated NSCs **(J,K)** and in differentiated NSCs **(L,M)**. Western bolts were normalized to the control samples. All values are presented as the mean ± SEM. *P < 0.05, **P < 0.01, ***P < 0.001 by one-way ANOVA. *n* = 3 independent experiments. **(A–D)** Scale bar, 50 μm.

To examine the relationship between hippocampal neurogenesis and mild hypoxia more directly, we conducted *in vitro* experiments with primary NSCs from fetal rat hippocampi. In proliferation assays, 5% hypoxic exposure increased the percentage of BrdU+ cells compared to normoxia, and the proliferative effect of hypoxia was blunted by concomitant DKK-1 treatment (Figures 6A,E), suggesting that Wnt pathway activation is required for mild hypoxia-induced NSC proliferation. We then examined mild hypoxia effects on neuronal differentiation of NSCs in neurobasal medium (Figure 6B). Hypoxia increased the proportion of NeuN+ cells and decreased that of GFAP+ cells (astrocytes). Blockade of Wnt signaling by DKK-1 treatment significantly reduced the proportion of NeuN+ cells (Figure 6F) but had no influence on GFAP+ cells (Figure 6G), suggesting that mild hypoxia induces NSC differentiation into neurons *via* activating Wnt pathway signaling. As expected, we also observed more cells immunopositive for the neural precursor marker DCX in hypoxia-treated cultures than in normoxic cultures.

Again, this effect was reversed by DKK-1 (Figures 6C,H), further demonstrating that hypoxia promotes neuronal differentiation through the Wnt pathway. In addition, hypoxia-treated cultures exhibited more Wnt3a+ cells than normoxic cultures, an effect also attenuated by DKK-1 (Figures 6D,I), confirming the requirement of Wnt pathway activation for hypoxia- induced NSC differentiation. Western blot analysis further indicated that mild hypoxia substantially increased the expression levels of Wnt3a, β-catenin, p-GSK-3β, and CyclinD1 in proliferating and differentiated NSCs, while DKK-1 treatment attenuated expression of these Wnt pathway proteins (Figures 6J–M). Taking together, we conclude that 5% hypoxia promotes proliferation and neuronal differentiation of hippocampal NSCs in part *via* Wnt pathway activation *in vivo* and *vitro*.

### Wnt/β-Catenin Pathway Blockade Inhibits CIHH-Induced Enhancement of Functional Synaptic Plasticity in Epileptic Rats

**Figure 7:**
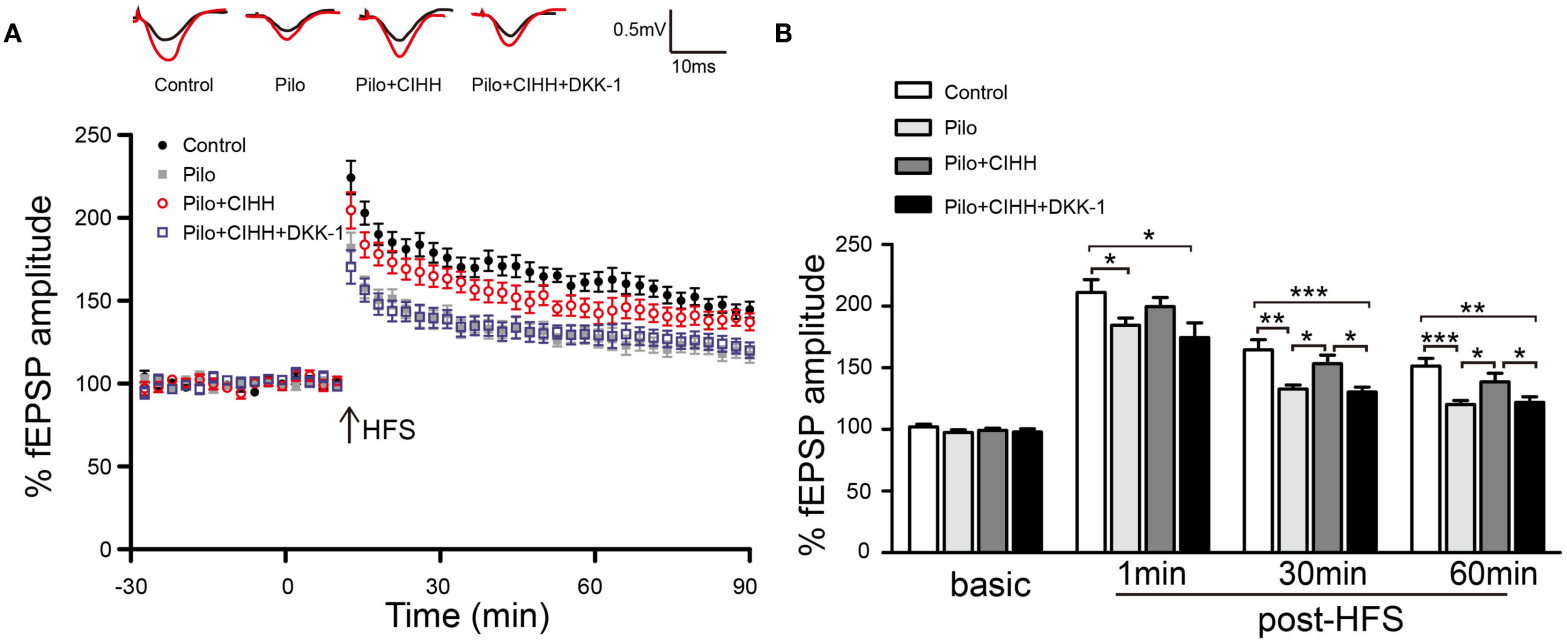
CIHH rescues epilepsy-induced LTP suppression through the Wnt/β-catenin pathway. **(A)** Normalized fEPSP amplitudes before and after high-frequency stimulation (HFS). **(B)** Histograms comparing mean fEPSP amplitudes at 1, 30, and 60 min post-HFS show LTP suppression by epilepsy and rescue by CIHH but not by CIHH + DKK-1. All values are presented as the mean ± SEM. *P < 0.05, **P < 0.01, ***P < 0.001 by ANOVA. *n* = 6 rats per group.

Finally, we investigated the influence of CIHH on synaptic plasticity and the contribution of Wnt pathway by measuring LTP at Schaffer collateral (SC)–CA1 synapses of rats injected with DKK-1 or vehicle. There were no differences in baseline fEPSP amplitude among groups, suggesting no effects on basal glutamatergic transmission. At 1, 30, and 60 min post-HFS, however, LTP was suppressed in epileptic (Pilo group) rats compared to the control group. CIHH alleviated LTP suppression at 30 and 60 min post-HFS, and this rescue was inhibited by DKK-1 (Figures 7A,B). Thus, CIHH mitigates the deleterious effects of epilepsy on functional plasticity at hippocampal SC–CA1 synapses, at least in part through activation of Wnt/β-catenin pathway.

## Discussion

We demonstrate that CIHH can reverse cognitive impairments caused by pilocarpine-induced epilepsy in rats, likely by rescuing deficits in hippocampal neurogenesis, structural neuronal plasticity, and functional synaptic plasticity through the Wnt/β-catenin signaling pathway. First, CIHH promoted hippocampal neurogenesis in epileptic rats and cell culture by increasing NSC proliferation and neuronal differentiation. Second, CIHH preserved dendritic morphological complexity and normal synaptic ultrastructure in epileptic rats. Finally, CIHH rescued long-term potentiation in epileptic rats.

Regulation of NSC proliferation and differentiation by hypoxia involves multiple signaling pathways, including Wnt/β-catenin (Mazumdar et al., 2010), Notch (Zhang et al., 2014; Man et al., 2018), and bone morphogenetic protein (BMP) (Pistollato et al., 2009). Blockade of Wnt pathway by direct injection of the Wnt/β-catenin antagonist DKK-1 into the adult hippocampus attenuated the reparative actions of CIHH on adult neurogenesis, synaptic plasticity, and hippocampus- dependent cognitive function. Collectively, these results strongly implicate Wnt pathway in the neuroprotective and nootropic effects of CIHH in epileptic rats.

CIHH has potential therapeutic value in several neurological disorders (Zhu et al., 2010; Varela-Nallar et al., 2014). In our study, hippocampus-dependent spatial learning and memory as measured in the MWM were impaired by epilepsy, consistent with previous report (Tavakoli et al., 2011), and CIHH effectively ameliorated these cognitive deficits. In addition, CIHH reversed hippocampal neuronal loss, reduced neurogenesis, and impaired synaptic plasticity, all of which are strongly associated with cognitive dysfunction (Zhang et al., 2014; Bouslama et al., 2015). Indeed, loss of neurons in hippocampal CA1 and CA3 subregions is a key pathological feature of TLE with cognitive impairment. Inhibition of epilepsy-induced neuronal loss in hippocampus likely resulted from preservation of hippocampal neurogenesis by CIHH. In the mammalian brain, NSCs exist in the subventricular zone (SVZ) and DG throughout life (Ming and Song, 2005; Thom et al., 2009), and maintenance of these populations and ensuing addition of newborn neurons to hippocampal circuits are considered crucial to learning and memory (Drew and Hen, 2007; Deng et al., 2010; Zhang et al., 2015). We found that the number of proliferating NSCs in the DG as indicated by BrdU immunostaining was reduced by epilepsy as previously reported (Hu et al., 2015) and rescued by CIHH. Many of these BrdU+ cells were also positive for the neuronal progenitor marker DCX and the mature neuronal marker NeuN, indicating enhanced neuronal differentiation in the epileptic hippocampus by CIHH.

In addition to neurogenesis, CIHH also rescued epilepsy-induced reductions in dendritic spine density and dendritic arbor complexity as revealed by Golgi staining and Sholl analysis. Epilepsy can result in abnormal neurogenesis and the development of aberrant dendrites and mossy fibers (Murphy et al., 2011), leading to further epileptogenic feedback excitation. However, this neurogenic response occurs mainly during the acute period, while hippocampal neurogenesis severely declines in the chronic phase associated with cognitive deficits (Helmstaedter et al., 2003; Hattiangady et al., 2004). The diversity of dendritic structures, including dendritic morphology and spine density, is the basis for implementing circuit functions during learning and memory (Hausser et al., 2000). Our research indicates that CIHH can partially mitigate deficient dendritic structural plasticity after epilepsy, including dendritic branches and spine density, but have no significant effect on dendritic length. The mechanism needs to be further studied. Rescue of structural plasticity was strongly associated with recovery of spatial memory performance. Moreover, we found that CIHH reversed abnormalities in synaptic ultrastructure and sustained normal expression of the plasticity-associated proteins Syt, PSD-95, and Kalirin-7 in the epileptic hippocampus.

The Wnt pathway is strongly implicated in hypoxia- mediated neuroprotection (Mazumdar et al., 2010; Cui et al., 2011; Zhang et al., 2011) and hippocampus- dependent learning and memory (Tabatadze et al., 2012; Fortress et al., 2013). When Wnt/β-catenin pathway is activated, Wnt ligand binds to the Frizzled (Fz) and its co-receptor to form a Wnt-Fz-LRP5/6 complex. Then the complex recruits the protein Disheveled (Dvl) and results in LRP5/6 phosphorylation, recruitment of Axin to the receptors and GSK-3β/APC/Axin separation (MacDonald et al., 2009). These events lead to increase of GSK-3β phosphorylation and inhibition of β-catenin phosphorylation (Logan and Nusse, 2004). β-catenin sequentially accumulates in cytoplasm and travels into the nucleus to activate Wnt target gene expression, such as CyclinD1, c-Myc, Axin2 (Toledo et al., 2008). In our study, hippocampal expression levels of Wnt3a and β-catenin were enhanced by CIHH treatment concomitant with cognitive improvement and neuroprotection. This result indicates that CIHH may activate Wnt/β-catenin signaling. Several endogenous ligands, such as the DKK family, Wnt inhibitory factor-1 (WIF-1) and the secreted Frizzled related proteins (sFRPs), act as antagonists of Wnt/β-catenin pathway (MacDonald et al., 2009). DKK-1, as a prototypical DKK family member, is the most important antagonist of the canonical Wnt signaling. It binds to the LRP5/6 receptor to prevent Wnt protein from forming a complex with LRP5/6 and Fz (Maguschak and Ressler, 2011; Scott and Brann, 2013) and then interferes the downstream gene expression. In previous researches, DKK-1 local injection in brain of animal models (Maguschak and Ressler, 2011; Scott and Brann, 2013) or DKK-1 addition into cell cultures (Killick et al., 2014; Tiwari et al., 2015) can both inhibit Wnt/β-catenin signaling *in vivo* and *vitro*. In our research, we used DKK-1 during CIHH treatment to examine the effect of Wnt/β-catenin signaling in CIHH or mild hypoxia. Indeed, hippocampal injection of the Wnt pathway inhibitor DKK-1 ameliorated Wnt3a and β-catenin upregulation as well as all observed benefits of CIHH on spatial learning and memory, object recognition memory, neurogenesis, and neuronal differentiation. Further, 5% hypoxia induced NSC proliferation and differentiation *in vitro*. This effect was specific to the neuronal lineage pathway as hypoxia enhanced the number of NeuN+ neurons and DCX+ neural progenitors but not GFAP+ astrocytes. Again, these effects were inhibited by Wnt pathway blockade, consistent with our *in vivo* findings. Accumulating evidence suggests that Wnt signaling is a principal regulator of adult hippocampal neurogenesis (Zhao et al., 2016). The Wnt pathway plays a critical role in the control of neuronal fate commitment and NSC proliferation (Lie et al., 2005). In addition, O_2_ availability has a direct effect on stem cell regulation *via* Wnt signaling (Mazumdar et al., 2010). Low O_2_ levels in the stem cell microenvironment may regulate adult neurogenesis by promoting NSC/progenitor cell proliferation and differentiation *via* Wnt signaling (Zhang et al., 2011), a notion supported by the current results. Moreover, generation of new neurons and integration into existing hippocampal circuits through synaptogenesis are essential for the maintenance of cognitive function in adults (Toni et al., 2008; Deng et al., 2010).

Synaptic plasticity underlies learning and memory formation in the mammalian brain (Whitlock et al., 2006; Nabavi et al., 2014). Long term potentiation, a possible cellular mechanism for contextual learning and memory (Brandwein and Nguyen, 2019), was used to evaluate functional synaptic plasticity as deficient LTP strongly correlates with learning deficits in epilepsy (Zhang et al., 2010). Intermittent hypoxia has been shown to regulate synaptic function and improve memory performance (Tsai et al., 2013), and current results demonstrated that CIHH effectively protected against epilepsy-induced LTP deficits concomitant with spatial and non-spatial memory improvements. Again, these effects of CIHH were inhibited by Wnt signaling blockade, in agreement with previous studies, suggesting an important role for the Wnt pathway in restoring the structure and function of synapses (Moon et al., 2004; Chen et al., 2006; Gupta and Schnell, 2019). Collectively, our data strengthen the notion that CIHH can reinstate normal synaptic connections and enhance synapse formation, stabilization and plasticity *via* Wnt signaling, thereby preserving hippocampal learning and memory in epilepsy.

## Conclusion

We report that neuronal loss, reduced neurogenesis, and impaired synaptic plasticity in the hippocampus of epileptic rats can be partly restored by CIHH through activation of the Wnt pathway. Our findings demonstrate the remarkable efficacy of CIHH against epilepsy-associated cognitive dysfunction and highlight Wnt/β-catenin pathway as a promising target for novel therapies to improve cognition in epileptic patients.

## Data Availability Statement

The original contributions presented in the study are included in the article/supplementary materials, further inquiries can be directed to the corresponding author/s.

## Ethics Statement

The animal study was reviewed and approved by the Second Hospital of Hebei Medical University.

## Author Contributions

JZ and WW conceived and designed the experiments. CS and JF performed the majority of the laboratory work, and participated in writing. ZQ analyzed the data. LJ and DL involved in writing. All authors read and approved the final manuscript.

## Conflict of Interest

The authors declare that the research was conducted in the absence of any commercial or financial relationships that could be construed as a potential conflict of interest.
